# Biological and technical complexities in analyzing extracellular vesicle immune interactions in B‐cell malignancies

**DOI:** 10.1002/1873-3468.70102

**Published:** 2025-07-04

**Authors:** Daniel Bachurski, Michael Hallek

**Affiliations:** ^1^ Department I of Internal Medicine Center for Integrated Oncology Aachen Bonn Cologne Duesseldorf, University of Cologne, Faculty of Medicine and University Hospital Cologne Cologne Germany; ^2^ Center for Molecular Medicine Cologne, University of Cologne Cologne Germany; ^3^ Cancer Research Center Cologne Essen (CCCE), Faculty of Medicine and University Hospital Cologne, University of Cologne Cologne Germany; ^4^ Mildred Scheel School of Oncology Aachen Bonn Cologne Düsseldorf, Faculty of Medicine and University Hospital of Cologne Cologne Germany; ^5^ CECAD Center of Excellence on Cellular Stress Responses in Aging‐Associated Diseases, University of Cologne Cologne Germany

**Keywords:** B‐cell malignancies, extracellular vesicles, single‐cell analysis, tumor microenvironment, chronic lymphocytic leukemia, EV labeling, TeLEV

## Abstract

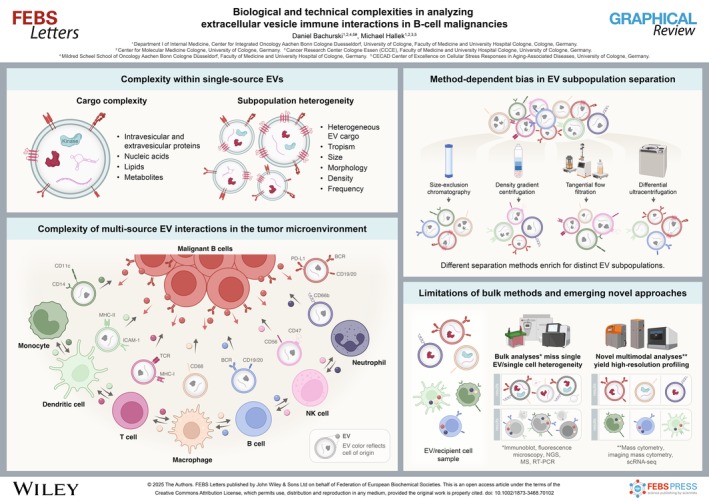

## Graphical abstract

Extracellular vesicles (EVs) mediate intercellular communication in tumor immune microenvironments. However, their role in B‐cell malignancies remains poorly defined, owing to biological complexity and technical challenges in EV isolation and analysis. Advancing EV and recipient cell analysis at the single‐EV and single‐cell level via novel tagging strategies is essential to overcome these obstacles. This graphical review examines the heterogeneity of EV cargo and subpopulation composition from diverse sources and their immune interactions in the tumor immune microenvironment of B‐cell malignancies, while discussing innovative methods for analyzing immune cell EVs and their target cells.

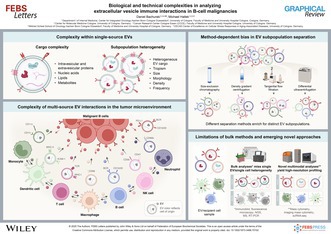

**Abbreviations**
BCR: B‐cell receptor
BTK: Bruton's Tyrosine Kinase
CAR T cell: Chimeric antigen receptor T‐cell
CLL: Chronic lymphocytic leukemia
DLBCL: Diffuse large B‐cell lymphoma
EV(s): Extracellular vesicle(s)
ICAM‐1: Intercellular adhesion molecule 1
ICP(s): immune checkpoint protein(s)
MISEV: Minimal information for studies of extracellular vesicles
sEV(s): Small EVs
SMLM: Single‐molecule localization microscopy
SP‐IRIS: Single‐particle interferometric reflectance imaging sensing
TIME: Tumor immune microenvironment

Extracellular vesicles (EVs), including exosomes and ectosomes, are critical mediators of intercellular immune communication and hold potential as diagnostic and therapeutic agents in oncology[1, 2]. Small EVs (sEVs), 50‐200 nm in size, carry a diverse array of intra‐ and extravesicular proteins, nucleic acids, metabolites, and lipids that drive biological functions[3, 4]. However, bulk analysis methods limit precise understanding, especially of immune cell EV compositional and functional heterogeneity in B‐cell malignancies, such as chronic lymphocytic leukemia (CLL) or diffuse large B‐cell lymphoma (DLBCL)[2, 5]. For instance, cell‐specific EV uptake and functional assays that use lipophilic labeling with flow cytometry or single‐cell imaging suffer from specificity issues and artifacts[5, 6].

Although current studies do not fully address the biological and technical complexities of EV and EV recipient cell analyses, the field has made considerable progress in revealing their involvement in a broad spectrum of physiological and pathological processes[7, 8]. Accordingly, advances in single‐EV and single‐cell analytical methods hold substantial potential[9]. Advanced multi‐dimensional techniques such as mass cytometry, imaging mass cytometry, and single‐cell RNA sequencing now enable profiling with over 40 dimensions to delineate EV‐mediated immune interactions within the tumor immune microenvironment (TIME) of B‐cell malignancies[10]. Novel multi‐modal EV tagging strategies must be developed to integrate these technology‐driven tumor microenvironmental insights with precise EV tracking and to determine EV effects on immune cell composition and states[11].

## The roles of immune cell EVs in the TIME of B‐cell malignancies

Immune cell EVs are critical mediators of intercellular communication in B‐cell malignancies, and although EVs released by malignant B cells have been characterized for their effects on fibroblasts, T cells, and myeloid cells, those produced by other immune cells subsets within the TIME remain poorly defined despite their major contributions to immune evasion and protumor signaling[12, 13, 14, 15]. These EVs carry molecular cargo that reflects the functional state of their parent cells[2]. In B‐cell malignancies increasingly treated with CAR T cell therapy, EVs from malignant B cells display surface markers such as CD19 and CD20, the B‐cell receptor, and its associated kinases[13, 16]. In contrast, EVs from CAR T cells can mediate cytotoxic effects on target cells from a distance[17]. Interactions involving receptor‐ligand pairs, such as CD11c on myeloid cell EVs and ICAM‐1 on other immune cell EVs, further underscore the complexity of immune cell EV‐mediated communication[18]. Notably, EVs released by malignant B cells and immune cells within the TIME are enriched in immune checkpoint proteins (ICPs), including PD‐L1, VISTA, and B7‐H2, which collectively contribute to tumor‐mediated immunosuppression through modulation of effector immune cell activity[13, 19, 20]. These EVs can reprogram bystander immune cells toward an immunosuppressive phenotype, in part by amplifying ICP expression[14]. A central mechanism involves the engagement of EV‐associated PD‐L1 with PD‐1 on T cells, leading to T cell exhaustion and functional impairment.

Additionally, EVs derived from T cells carrying T‐cell receptors interact with peptides bound to MHC class II complexes on EVs released by antigen‐presenting cells. We hypothesize that these EV‐based immune cell‐specific surface protein complexes can be transferred to recipient cells, resulting in a dynamic and adaptable surface proteome responsive to shifts in the TIME.[21].

## The complexity and function of single‐EV cargo can be delineated by multi‐modal EV‐tagging strategies

EV cargo such as kinases, lipids, metabolites, and nucleic acids can act in synergistic or antagonistic fashions to modulate recipient cell phenotypes in a manner dependent on cell context[3, 22]. Individual EV components’ functional contributions and interactions remain poorly defined, as evidenced by ongoing debates over microRNA uptake and efficacy. This gap in knowledge necessitates comprehensive and innovative investigations at high‐dimensional single‐cell resolution to elucidate EV function and cargo dynamics fully, as conventional approaches using lipophilic dyes and genetic fusion proteins are inadequate for patient cell systems[23]. Multi‐modal EV‐tagging strategies, including protein‐based tags, lipophilic dyes, and RNA‐based tags, allow for characterizing EV heterogeneity and enable comprehensive tracing of distinct EV components. However, each tagging method has inherent limitations. Lipophilic dyes, although widely applicable, may produce nonspecific staining and false‐positive results due to non‐EV aggregates[6]. Protein‐based tags, such as genetically encoded fluorescent fusion proteins (e.g., CD63‐GFP), can bias analyses toward specific EV subpopulations and are limited by low transfection or transduction efficiency in primary cell models[24]. RNA‐based tags provide specificity for RNA cargo but are prone to interference from nucleic acid contaminants or nonspecific extracellular RNA associations[25]. To mitigate these issues, we recommend an integrative multi‐modal tagging approach that combines the complementary strengths of each method.

## EV subpopulations are heterogeneous in composition and function

EV subpopulation heterogeneity is a rapidly emerging focus in the field, now being addressed by state‐of‐the‐art single‐EV techniques, such as nanoparticle tracking analysis, nanoflow cytometry, single‐particle interferometric imaging sensing (SP‐IRIS), Raman spectroscopy, or high‐resolution single‐molecule localization microscopy (SMLM)[26, 27]. Previous studies relied on bulk analyses of EVs and EV recipient cells, which overlook the existence of EV subpopulations carrying distinct biologically active cargo. This hampers a deeper understanding of the functional diversity within EV uptake and function[27]. We highlight the importance of exploring established disease‐related proteins and pathways, such as the B‐cell receptor and interleukin receptor signaling pathways, or ICPs, for their potential involvement in EV cargo sorting, release, and functional effects on recipient cells[28, 29]. Such investigations may reveal novel mechanisms operating within the TIME and guide innovative therapeutic strategies targeting this EV‐shaped microenvironment[30]. In particular, Bruton's tyrosine kinase (BTK) represents an interesting example due to its known role in EV release in CLL cells[31, 32].

## EV separation methods enrich distinct EV subpopulations

Established EV separation methods, including size‐exclusion chromatography, density gradient centrifugation, tangential flow filtration, and differential ultracentrifugation, are fundamental for investigating EV effects *in vitro* and *in vivo*. However, these approaches yield EVs of differing quality and quantity[33]. When enriching EVs from liquid biopsies, distinct subpopulations may be selectively isolated, or EVs from multiple sources redistributed, potentially resulting in loss or artificial enrichment of specific immune cell‐derived subtypes[34]. The MISEV guidelines provide a framework for quality controls to assess the impact of these separation and concentration methods[35].

## Bulk EV and EV recipient analyses miss single‐EV and single‐recipient cell heterogeneity

Current bulk analyses fail to capture the heterogeneity of EV subpopulations, uptake dynamics, and single‐recipient cell heterogeneity[36]. Comprehensive characterization of EV contributions to the TIME requires multi‐omic approaches at single‐EV and single‐cell resolution, necessitating the development of novel EV labeling strategies based on the EV proteome, transcriptome, and metabolome[37, 38].

## Conclusion

The emerging field of EVs offers novel insights into intercellular communication within the TIME of B‐cell malignancies. However, inherent biological and technical complexities demand methodological advances and rigorously controlled experiments to address EV cargo and subpopulation heterogeneity, particularly given the diverse EV immune interactions that influence tumor microenvironmental composition in lymphoid malignancies. Together with completing the characterization of key EV‐mediated mechanisms, the rapid advancement of the EV field presents a compelling opportunity to unlock the full translational potential of EVs as therapeutics and diagnostics in B‐cell malignancies.

## Author contributions

DB and MH jointly conceived, designed, and prepared the graphical review and manuscript.
